# Increased Peripheral Blood Pro-Inflammatory/Cytotoxic Lymphocytes in Children with Bronchiectasis

**DOI:** 10.1371/journal.pone.0133695

**Published:** 2015-08-10

**Authors:** G. Hodge, J. W. Upham, A. B. Chang, K. J. Baines, S. T. Yerkovich, S. J. Pizzutto, S. Hodge

**Affiliations:** 1 Lung Research, Hanson Institute and Dept. Thoracic Medicine, Royal Adelaide Hospital, Adelaide, Australia; 2 The University of Queensland School of Medicine, Princess Alexandra Hospital, Brisbane, Queensland, Australia; 3 Child Health Division, Menzies School of Health Research, Darwin, Northern Territory, Australia; 4 Department of Respiratory and Sleep Medicine, Lady Cilento Children’s Hospital, Queensland University of Technology, Children’s Health Queensland, South Brisbane, Queensland Australia; 5 Dept. Respiratory and Sleep Medicine, Hunter Medical Research Institute, Newcastle, New South Wales, Australia; 6 University of Newcastle, New South Wales, Australia; 7 The University of Queensland School of Medicine and Prince Charles Hospital, Brisbane, Queensland, Australia; University of Modena and Reggio Emilia, ITALY

## Abstract

**Objective:**

Bronchiectasis (BE) in children is common in some communities including Indigenous children in Australia. Relatively little is known about the nature of systemic inflammation in these children, especially the contribution of specific pro-inflammatory and cytotoxic lymphocyte subsets: T-cells, natural killer (NK) cells and NKT-like cells. We have shown that these cells produce increased cytotoxic (granzyme b and perforin) and inflammatory (IFNγ and TNFα) mediators in several adult chronic lung diseases and hypothesised that similar changes would be evident in children with BE.

**Methods:**

Intracellular cytotoxic mediators perforin and granzyme b and pro-inflammatory cytokines were measured in T cell subsets, NKT-like and NK cells from blood and bronchoalveolar samples from 12 children with BE and 10 aged-matched control children using flow cytometry.

**Results:**

There was a significant increase in the percentage of CD8+ T cells and T and NKT-like subsets expressing perforin/granzyme and IFNγ and TNFα in blood in BE compared with controls. There was a further increase in the percentage of pro-inflammatory cytotoxic T cells in Indigenous compared with non-Indigenous children. There was no change in any of these mediators in BAL.

**Conclusions:**

Childhood bronchiectasis is associated with increased systemic pro-inflammatory/cytotoxic lymphocytes in the peripheral blood. Future studies need to examine the extent to which elevated levels of pro-inflammatory cytotoxic cells predict future co-morbidities.

## Introduction

Bronchiectasis (BE) is a progressive disease of the airways characterised by chronic infection and associated inflammation. Although uncommon in children living in developed countries, BE is common in some communities such as in Indigenous children in Alaska (USA) and Australia [1]. In these populations where indigenous groups experienced poorer housing and sociodemographic circumstances, BE is present in one in every 63–68 children and often begins very early in life [2] where prematurity and increased frequency and earlier onset of acute lower respiratory infections is more common than in non-Indigenous groups [2]. Poor and inadequate treatment leads to loss of lung function and subsequent reduction in life expectancy [2], whereas effective management of BE has been shown to reduce morbidity [3].

The potential links between long-term inflammation and co-morbidities such as the development of dysplasia and cancer [4,5], microbial infection [6] and cardiovascular disease [7] have been well described. In this regard there have been reports of increases in systemic markers of inflammation during stable phases of BE in adult patients [8], and BE has been shown to be an independent risk factor for atherosclerosis [9] cardiovascular disease, which is 1.3 times more common in Indigenous people, and osteoporosis, which is 1.8 times more common in Indigenous males than their non-Indigenous counterparts [10]. The lower sociodemographic conditions experienced by Indigenous groups is likely to contribute to a higher incidence of systemic disease, including the increased rate of BE as well as increased colonisation of the lower airway with microorganisms including non-typeable *Haemophilus influenzae* (NTHi) and *S*. *pneumoniae*.

Our group has reported increased production of Th1 pro-inflammatory cytokines IFN-γ and TNF-α by CD8+ T cells in both peripheral blood and lungs [11] and higher levels of the cytotoxic mediators granzyme b and perforin in peripheral blood NKT-like and NK cells in adult inflammatory lung disease including chronic obstructive pulmonary disease (COPD), severe asthma and bronchiolitis obliterans syndrome following lung transplantation [12–15]. However relatively little is known about the nature of systemic inflammation in BE. Some limited information suggests systemic cytokine over-production and neutrophilic inflammation in BE [16], but it is not known whether the systemic inflammation also involves lymphocyte populations in the circulation.

We hypothesized that these markers of systemic inflammation may also be present in children with BE, especially in Indigenous children where BE is generally more severe when diagnosed [1]. To investigate this hypothesis, we determined whether T, NKT-like and NK cells from children with BE express increased levels of cytotoxic mediators, perforin and granzyme b and pro-inflammatory cytokines in peripheral blood and airways compared with aged matched children without BE.

## Materials and Methods

### Patient and control groups

Children were recruited from the Royal Children’s Hospital in Brisbane and the Royal Darwin Hospital. The study was approved by the Institutional Ethics Committee from both hospitals and written informed consent obtained from each parent or carer. Inclusion criteria included the diagnosis of BE. Aged matched control children were undergoing bronchoscopy with no history of chronic cough or wheeze eg., evaluation of stridor or re-evaluation of structural airway abnormality. Exclusion criteria included a clinical history of primary aspiration (coughs or chokes with at least every second feed), neuromuscular problems, primary adaptive immunity abnormality, past history of invasive infections (meningitis etc), recurrent (>2) pneumonia and other respiratory disease. All children had a standardized medical history taken with a focus on respiratory history including cough quality (wet/dry) [17]. Demographics collected included possible contributors to frequency of respiratory infections (day care attendance, smoke exposure, number of siblings in house-hold, socioeconomic status, vaccinations and recent use of antibiotics). Sweat test for exclusion of cystic fibrosis was performed as well as spirometry for those aged >4 years. Blood and BAL was obtained during bronchoscopy (performed routinely under general anesthesia) [18] and microbiology determined on BAL. All children were in stable state (ie no current exacerbation) when the specimens were collected. We investigated respiratory bacteria and viruses of clinical significance in the BAL of children in this study, including mycoplasma, influenza, Adenovirus, *Strep*. *pneumonia*e, *Staph aureus*, *Moraxella catatthalis*, *Pseudomonas aeruginosa* and *H*. *influenza*e.

### Leukocyte numbers in peripheral blood and BAL

Full blood counts, including white cell differential counts, were determined on blood specimens using a CELL-DYN 4000 (Abbot Diagnostics, Sydney, Australia). BAL cell counts were determined using a haemocytometer. Blood films and BAL cytospins were stained by the May-Grunwald-Giemsa method and white cell differential counts checked by morphological assessment microscopically.

### CD4/CD8 T, NKT-like and NK cell percentages

CD4 and CD8 T, NKT-like and NK cell percentages in blood and BAL were enumerated as previously reported using flow cytometry [11–13].

### Perforin and granzyme b expression in T, NKT-like and NK cells

Expression of perforin and granzyme b was also determined in T, NKT-like and NK cells as previously reported [11–13]. Briefly, BAL was centrifuged 300 ×g for 5 min and cells re-suspended at 5x10^5^ mL in RPMI 1640 medium (Gibco, New York, USA) supplemented with 125 U/mL penicillin and 125 U/mL streptomycin (Gibco). Aliquots of 150 μL blood and 100 μL BAL were added to 2 mL FACSLyse (BD Biosciences, Sydney, Australia) for 10 min at room temperature. Cells were then centrifuged 300 ×g for 5 min and the cell pellet re-suspended in 0.5 mL FACSPerm (BD) for 10 min at room temperature. Two mL 0.5% bovine serum albumin (Sigma/Aldrich, Sydney, Australia) / Isoflow (Beckman Coulter, Sydney, Australia) was then added and the tubes centrifuged at 300 ×g for 5 min. Five μL of appropriately diluted anti- perforin FITC (eBioscience, Sydney, Australia), CD3 perCP.CY5.5 (BD), CD4 PE.CY7 (BD), CD56 APC (Beckman Coulter, Sydney, Australia), CD8 APC.CY7 (BD), granzyme b V450 (BD) and CD45 V500 (BD) monoclonal antibodies were added for 15 min in the dark at room temperature for 15 min. Two mL of 0.5% bovine serum albumin (Sigma) / Isoflow (Beckman Coulter) was then added and the tubes centrifuged at 300 × g for 5 min. After decanting, cells were analyzed within 1 h on a FACSCanto II flow cytometer using FACSDiva software (BD). Samples were analyzed by gating lymphocytes using CD45 staining versus side scatter (SSC). A minimum of 350,000 low SSC events for blood and 50,000 low SSC events for BAL were acquired in list-mode format for analysis. T cells were identified as CD3+CD56-CD45+ low FSC/SSC events, NKT-like cells were identified as CD3+CD56+CD45+ low FSC/SSC events and NK cells as CD3-CD56+ CD45+ low FSC/SSC events. Results are expressed as a percentage of lymphocytes.

### IFNγ and TNFα production by T, NKT-like and NK cells

Production of IFNγ and TNFα by T, NKT-like and NK cells was determined on blood and BAL samples from all subjects as previously reported [11–13].

Briefly, one mL blood diluted 1:2 in RPMI and prepared BAL cells were stimulated with phorbol myristate (25 ng/mL) (Sigma, Sydney, Australia) and ionomycin (1 μg/mL) (Sigma). Brefeldin A (10 μg/mL) was added as a “Golgi block” (Sigma) and the tubes re-incubated in a humidified 5% CO_2_/95% air atmosphere at 37°C for 16 h. Aliquots of blood and BAL were added to FACS tubes (BD) and treated with FACSLyse and FACSPerm as above and five μL of appropriately diluted anti- IFNγ FITC (BD), CD3 perCP.CY5.5 (BD), CD56 APC (Beckman Coulter, Sydney, Australia), CD8 APC.CY7 (BD), TNFα V450 (BD) and CD45 V500 (BD) monoclonal antibodies were added for 15 min in the dark at room temperature. Two mL of 0.5% bovine serum albumin (Sigma) / Isoflow (Beckman Coulter) was then added and the tubes centrifuged at 300 ×g for 5 min. After decanting, cells were analyzed as above.

### Statistical Analysis

Power analysis was performed based on our previous studies (eg, n = 12 allows a power of 92% to detect a 20% increase in perforin production by T-cells in peripheral blood). Statistical analysis was performed using the Mann-Whitney test. Bivariate correlation analyses between cell types, inflammatory/cytotoxic markers and Indigenous status, age, gender and viral/bacterial isolation from BAL, were performed using Spearman Rho correlation tests. SPSS software was applied and differences between groups of p<0.05 considered significant.

## Results

### Demographic details of subjects

The demographic details of the children studies are presented in Table 1. There were no significant differences in gender between groups. The BE group contained more Indigenous children compared to controls. There were non-significant trends (p = 0.069 and p = 0.079 respectively) for increased percentages of neutrophils in BAL and blood from children with BE compared to controls (Table 1).

**Table 1:** Demographic details of the children tested.

	Controls	Bronchiectasis
N	10	12
Age (months)	40 (19.5,83.3)	36 (27.82.5)
Gender (M/F)	7/3	5/7
Indigenous (Y/N)	1/9	6*/6
Blood WCC (x10^9^/L)	8.1 (7.3,10.0)	9.3 (8.2,11.0)
BAL WCC (x10^6^/L)	200 (126,234)	265 (152,360)
Blood neutrophils	2.5 (2.1,2.7)	3.2 (2.7,3.6)
CRP	2.0 (2,2)	2.0 (2,2)
BAL neutrophils	5.5 (2.8,8.0)	12.25 (6.8,25)
Mycoplasma (n)	0	0
Influenza (n)	0	0
RSV (n)	0	1
Adenovirus (n)	1	1
Power of 10 Bacteria cfu/mL)	3.5 (13,4)	4.0 (3,4)

*p<0.05 vs. control.

Data presented as median (q1, q3) unless otherwise indicated. N, number; WCC, total leucocyte count; BAL, bronchoalveolar lavage fluid; RSV, Respiratory syncytial virus; cfu, colony forming units.

### Organisms identified in BAL of children

Adenovirus was detected in BAL from 1 control and 1 child with BE, and RSV from one child with BE (Table 1). There was a range of organisms isolated from the BAL of both patient and control groups including *Streptococcus pneumonia*, *Staphylococcus aureus*, *Pseudomonas aeruginosa* and *Moraxella catarrhalis*. NTHi was detected from 50% of the children with BE vs. 10% of controls. Overall, the median (iq 1,3) number of organisms (power of 10 cfu/mL) for control children was 3.5 (3,4), and 4.00 (3,4) for children with BE, and no significant difference between the groups was noted (Table 1).

### CRP levels in blood

There were no differences in CRP levels between patient and control groups (Table 1).

### T, NKT-like and NK cell percentages

There was a significant increase in CD8+ T cells in the blood of children with BE compared with controls but no other differences in the percentages of T, CD4+T cells, NKT-like or NK cells in blood or BAL between groups (as a percentage of CD45+ lymphocytes) (Table 2).

**Table 2:** The percentages of T, CD8+T, NKT-like and NK cells in the blood and BAL of children with bronchiecstasis (BE) and controls. Data presented as median (q1, q3) unless otherwise indicated. There was a significant increase in the percentage of CD8+T cells in the blood of children with BE compared with control group (p = .010).

		T	CD8	NKT-like	NK
Blood	Control	73.5 (70.5,83.5)	25 (23,32)	1 (1,4)	6 (3,10)
	BE	84.5 (69.3,88)	**38 (31,48)** *	2 (1,3)	9 (6,16)
BAL	Control	79.9±12.6	63.5±14.9	6.3±3.8	5.9±4.1
	BE	80.9±13.1	53.0±13.6	7.7±6.5	4.1±2.9

*p<0.05 vs. control.

### Perforin and granzyme b expression in T, NKT-like and NK cells

**Fig 1:**
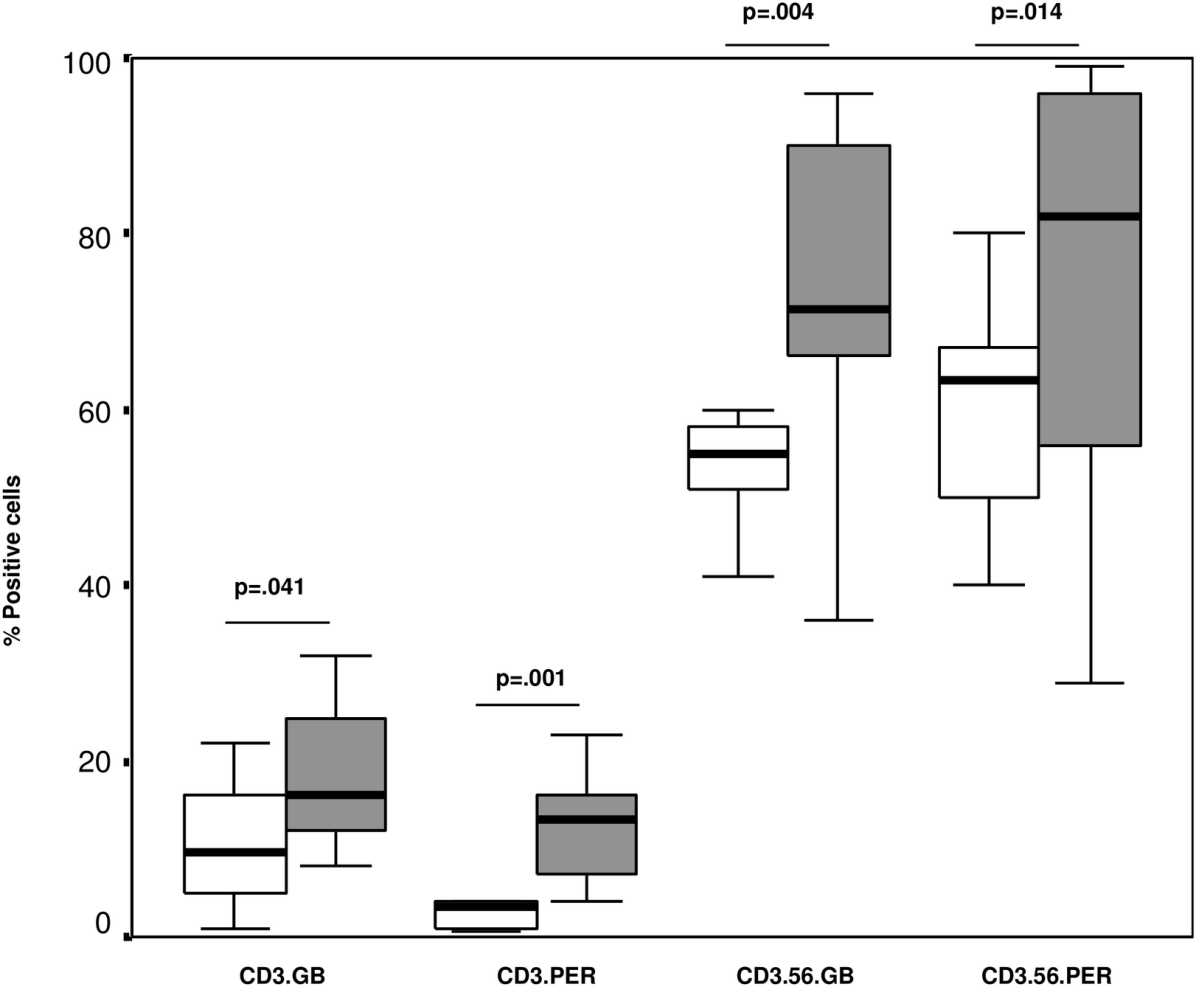
The percentages of T (CD3) and NKT-like (CD3.56) cells expressing granzyme b (GB) and perforin (PER) in the blood of children with BE (grey bars) compared with controls (clear bars). There was a significant increase in the percentages of T and NKT-like cells expressing granzyme b and perforin in the blood of children with BE compared with controls.

There was a significant increase in the percentages of T and NKT-like cells expressing granzyme b and perforin in the blood of children with BE compared with controls (Fig 1) but no difference in the percentage of NK cells expressing granzyme b and perforin between groups (granzyme b: 78.0±17.3 (74.3±11.2), perforin: 81.0±13.4 (86.7±10.3) for BE (Controls) respectively p>0.05 for all).

There was no significant differences in the percentages of T, NKT-like or NK cells expressing granzyme b or perforin in the BAL of children with BE compared with controls (Table 3).

**Table 3:** The percentages of T, NKT-like or NK cells expressing granzyme b (GB) and perforin (PER) in the BAL of children with BE compared with controls. There was no differences in the percentages of T, NKT-like or NK cells expressing granzyme b or perforin in the BAL of children with BE compared with controls (p>0.05 for all).

	T		NKT-like		NK	
	GB	PER	GB	PER	GB	PER
Control	4.5±12.6	1.1±0.7	8.1±2.9	3.3±2.2	3.6±2.9	1.7±0.9
BE	3.6±12.6	1.3±0.8	6.2±3.7	4.8±3.1	3.6±2.6	1.3±0.8

### IFNγ and TNFα production by T, NKT-like and NK cells

**Fig 2:**
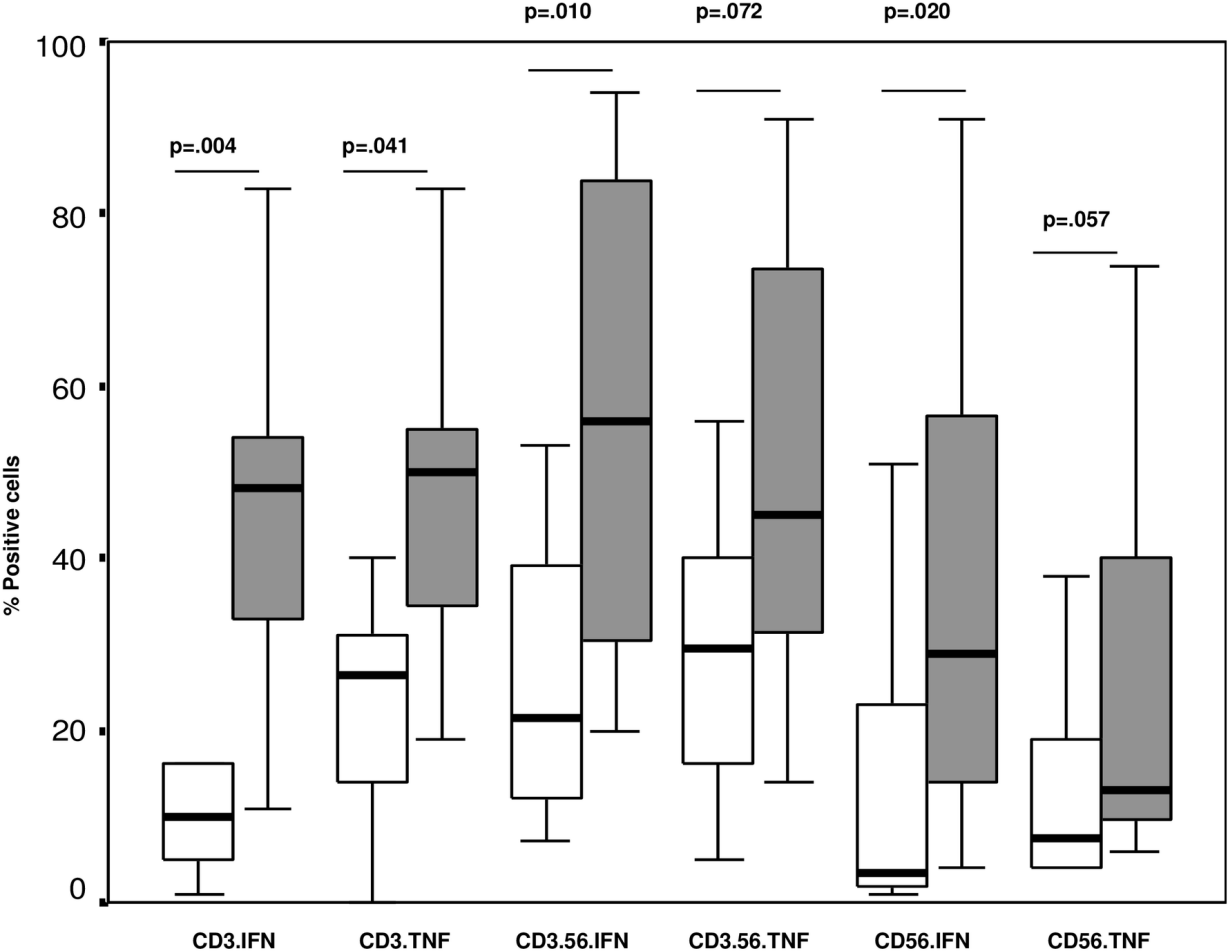
The percentages of T (CD3) and NKT-like (CD3.56) and NK cells (CD56) producing IFNγ and TNFα in the blood of children with BE (grey bars) compared with controls (clear bars). There was a significant increase in the percentages of T cells producing IFNγ and TNFα, NKT-like cells producing IFNγ (trend for TNFα) and NK cells producing IFNγ (trend for TNFα) in the blood of children with BE compared with controls.

There was a significant increase in the percentages of T cells producing IFNγ and TNFα, NKT-like cells producing IFNγ (trend for TNFα) and NK cells producing IFNγ (trend for TNFα) in the blood of children with BE compared with controls (Fig 2).

There was no significant differences in the percentages of T, NKT-like or NK cells producing IFNγ and TNFα in the BAL of children with BE compared with controls (Table 4).

**Table 4:** The percentages of T, NKT-like or NK cells producing IFNγ and TNFα in the BAL of children with BE compared with controls There was no significant differences in the percentages of T, NKT-like or NK cells producing IFNγ and TNFα in the BAL of children with BE compared with controls (p>0.05 for all).

	T		NKT-like		NK	
	IFNγ	TNFα	IFNγ	TNFα	IFNγ	TNFα
Control	25.5±8.5	24.2±9.3	33.7±10.1	29.0±7.8	11.8±6.6	14.8±7.7
BE	21.8±7.4	22.3±8.3	28.9±9.9	21.9±11.2	11.7±5.4	14.1±7.8

### Perforin/granzyme b expression and IFNγ/TNFα production in T, NKT-like and NK cells from Indigenous and non-Indigenous children with BE

To elucidate any differences in cytotoxic or pro-inflammatory lymphocyte subsets between Indigenous and non-Indigenous children with BE, we analysed granzyme b and perforin expression and IFNγ and TNFα production in lymphocyte subsets from these patient groups.

**Fig 3:**
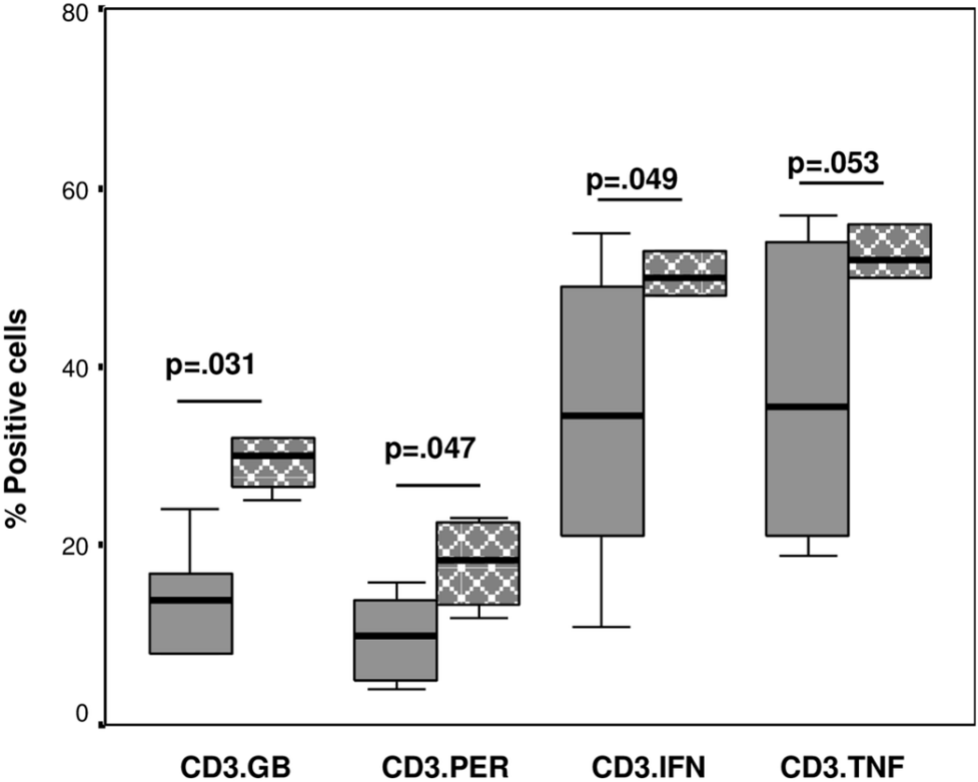
The percentages of T (CD3) expressing granzyme b (GB) and perforin (PER) and T cells producing IFNγ and TNFα in the blood of Indigenous children with BE (grey checkered bars) compared with non-Indigenous children with BE (grey bars). There was a significant increase in the percentages of T cells expressing granzyme b and perforin in the blood of Indigenous children with BE compared with non-Indigenous children with BE. There was a significant increase in the percentage of T cells producing IFNγ (trend for TNFα), in the blood of Indigenous children with BE compared with non-Indigenous children with BE.

There was a significant increase in the expression of granzyme b and perforin in T cells from Indigenous children with BE compared with non-Indigenous children (Fig 3). There was a significant increase in the percentage of T cells producing IFNγ (trend for TNFα) from Indigenous children with BE compared with non-Indigenous children (Fig 3).

**Fig 4:**
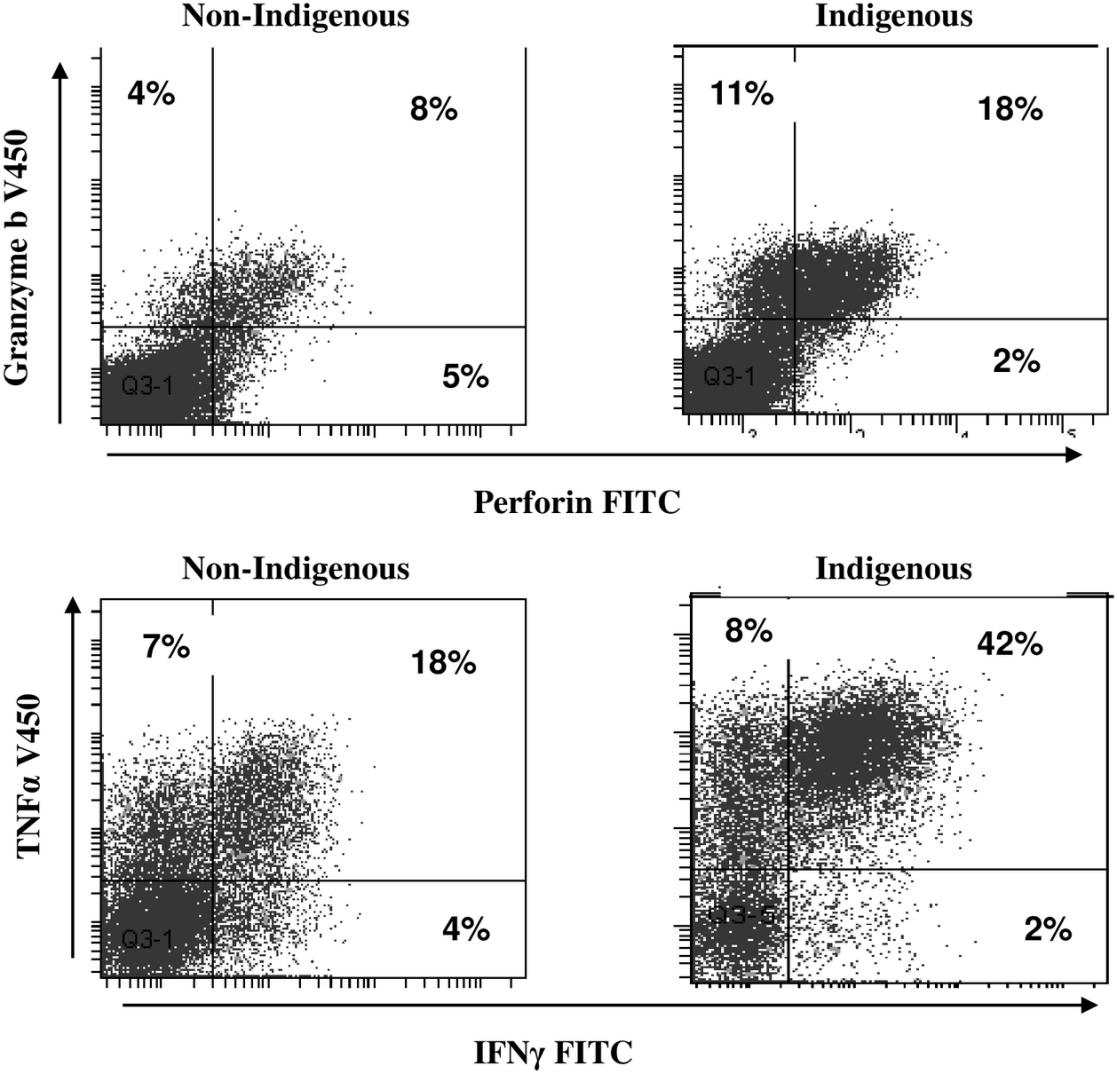
Representative flow cytometry plots of T cells (CD3+CD56) expressing granzyme b and perforin and producing IFNγ and TNFα in the blood of Indigenous children with BE compared with non-Indigenous children withy BE. There was a significant increase in the expression of granzyme b and perforin in T cells from Indigenous children with BE compared with non-Indigenous children. There was a significant increase in the percentage of T cells producing IFNγ (trend for TNFα) from Indigenous children with BE compared with non-Indigenous children.

There were no differences in the percentages of NKT-like or NK cells expressing granzyme b or perforin or producing IFNγ and TNFα in the blood of Indigenous children with BE compared with non-Indigenous children (p>0.05 for all) (data not shown). There were no differences in the percentages of NKT-like or NK cells expressing granzyme b or perforin or producing IFNγ and TNFα in the BAL of Indigenous children with BE compared with non-Indigenous children (p>0.05 for all) (data not shown). Representative flow cytometry plots of T cells expressing granzyme b and perforin and producing IFNγ and TNFα in the blood of Indigenous children with BE compared with non-Indigenous children with BE is shown in Fig 4.

### Bivariate correlation analysis

There were no significant associations between gender, age, Indigenous status, NTHi or adenovirus in BAL, T-cell numbers, and any of the inflammatory/cytotoxic parameters tested. A positive correlation was noted between *S*. *pneumoniae* isolation from BAL, and IFN-g and TNF-a production by T-cells (correlation coefficients .437 and .407; p = 0.033 and.049 respectively) and with NKT-cell production of IFN-g (correlation coefficients .460 p = 0.024). The percentage of CD8+ T-cells in blood correlated positively with T-cell production of both IFN-g and TNF-a (correlation coefficients .504 and .423; p = 0.010 and.040 respectively). NK numbers correlated with T-cell expression of perforin, granzyme b, IFN-g and TNF-a (correlation coefficients .505, .496, .460,.540; p = 0.014, 0.016,0.021,0.006 respectively). Numerous significant correlations were noted between the various inflammatory/cytotoxic mediators tested (Table 5).

**Table 5:** Bivariate analysis of correlations between inflammatory/cytotoxic mediators in blood.

	NKT	NK	Perforin T	Perforin NKT	Perforin NK	IFNγ T	IFNγ NKT	IFNγ NK	TNFα T	TNFα NKT	TNFα NK
Granzyme b T	0.241 (0.268)	0.486 **(0.019)** *	0.771 **(0.001)** *	0.203 (0.353)	0.631 (0.106)	0.405 (0.063)	0.482 **(0.027)** *	0.379 (0.090)	0.525 **(0.015)** *	0.550 **(0.010)** *	0.449 **(0.041)** *
Granzyme b NKT	0.309 (0.151)	0.074 (0.736)	0.207 (0.342)	0.326 (0.129)	0.654 **(0.001)** *	0.123 (0.566)	0.230 (0.315)	0.401 (0.072)	*0*.*130 (0*.*573)*	0.231 (0.313)	0.439 **(0.046)** *
Granzyme b NK	0.267 (0.217)	0.337 (0.116)	0.551 **(0.006)** *	0.756 **(0.000)** *	0.428 **(0.042)** *	0.269 (0.227)	0.503 **(0.020)** *	0.518 **(0.016)** *	0.177 (0.442)	0.434 **(0.049)** *	0.660 **(0.001)** *

Data presented as Pearson Correlation coefficient (p value. (2-tailed)).

*Significant correlations are highlighted in bold.

## Discussion

This is the first study to show that childhood BE is associated with increased peripheral blood cytotoxic and pro-inflammatory T, NKT-like and NK cells. The groups of children included in the present study characteristically have a high rate of generational cultural diversity; thus, determination of the influence of Indigenous status on the systemic immune response is difficult. Despite this, we found that children in the BE group that were identified by their parent as having Indigenous Australian ancestry, had a further increase in cytotoxic pro-inflammatory T cells in the peripheral blood compared with non-Indigenous children with BE. A potential limitation of this study is that a comparison between affected children and control children with the same ethnic component was not possible. However, our previous extensive investigations have shown that Indigenous status in children with lung disease has no significant effect with regards to systemic inflammation, suggesting that the differences noted in BE between Indigenous and non-Indigenous populations were not influenced by systemic inflammation. In a previous investigation of children with BE involving Indigenous and non-Indigenous children, we found that, using regression analysis, Indigenous descent was not an independent predictor of IFN-γ production [19]. We have also found that high pulmonary expression of IL-6 and IL-1β in children with chronic suppurative lung disease is associated with impaired recall responses to non-typeable *Haemophilus influenzae* (unpublished data: manuscript under consideration), Our analyses also found no difference between Indigenous and non-Indigenous children with respect to the degree of systemic inflammation measured by levels of IL-6, IL-8 and CRP. These novel findings of increased systemic cytotoxic pro-inflammatory T cells in children with BE, independent of Indigenous status, identifies an up-regulated systemic adaptive immune response and may reflect a more severe disease when BE is diagnosed [1].

Up to 50% of subjects with COPD have evidence of BE on computer tomography scanning [20]. Like COPD, BE has been shown to be associated with increases in systemic markers of inflammation and we hypothesized that we may elucidate similar findings in childhood BE, particularly in Indigenous children where BE is often more severe [1]. However, the systemic inflammation and pathogenic mechanisms that are present in the children with BE are likely to be very different to the systemic inflammation present in COPD. Although COPD is sometimes associated with BE in older adults (who also have cardiovascular comorbidities), COPD is a disease which manifests in older adults, who are likely to have altered immunological responses due to aging and smoking. Contributors to the systemic inflammation in children with BE may include a larger proportion of these children who had been exposed to cigarette smoke (66%) compared to control children (25%), recurrent lower respiratory tract infections and colonisation of the airway with microbes including *S*. *pneumoniae* and non-typeable *H*. *influenza*. In this regard we found that NTHi was isolated from 50% of the children with BE vs. 10% controls, and that there was a significant correlation between the presence of *S*. *pneumoniae* in BAL and the production of TNF-alpha and IFN-gamma by T and NKT-like cells in the children with BE.

Systemic changes noted in chronic lung disease have been suggested to result from a spill-over of inflammatory lymphocytes from the lung [21]. In this regard we have previously shown increased pro-inflammatory T, NKT-like and NK cells in the airways (BAL derived), peripheral blood and intraepithelial cells (bronchial brushings derived) from adults with chronic lung diseases including COPD and bronchiolitis obliterans syndrome following lung transplantation [12–15]. Interestingly, although we found similar changes in cytotoxic/pro-inflammatory lymphocyte subsets in the peripheral blood in children with BE, we did not detect these changes in the BAL, reflecting potentially different immunological response in patients with BE compared with COPD, although the possibility of a type II error due to BAL numbers cannot be excluded. There has been a report of increased activated CD8+ T cells infiltrating the bronchial wall below the basement membrane in adult patients with BE [20]. As such, a further study examining the cytotoxic/pro-inflammatory profile of intra-epithelial lymphocytes obtained by bronchial brushings from children with BE, as we have previously done for COPD patients, could prove enlightening [12,13].

There have been reports of elevated WCC, CRP, fibrinogen levels and bacterial colonization, suggesting the presence of a systemic inflammatory response, in clinically stable adult BE patients [22]. To our knowledge this is the first report of elevated systemic lymphocyte markers in childhood BE and raises the question of whether these cells that are active early on in life, are the instigators of co-morbid diseases prevalent in this patient group, later on in life. In this regard, cytotoxic pro-inflammatory CD8+ T and NK cells have been shown to be associated with atherosclerosis and osteoporosis in adults [23–25]. Although there was no correlation between the percentage of lymphocyte subsets expressing cytotoxic/pro-inflammatory mediators and other markers of systemic inflammation such as WCC, CRP or circulating neutrophil levels, this may be reflective of the age of the study group and disease duration and other markers such as ESR, hsCRP and HR-CT score may have proven useful and would be a worthwhile addition to future studies.

We and others have shown a correlation between soluble and T cell expression of TNFα and lung function as measure by forced expiry volume in one second (FEV1) in adult patients with COPD [12,24]. TNFα has been described as the possible driving force behind COPD [26]. The report that high plasma concentrations of TNFα were associated with severity of BE in clinically stable adult [16] patients may also suggest a possible role of TNFα in the pathogenesis of BE and longitudinal monitoring of lymphocyte subset pro-inflammatory profiles may be a useful tool in assessing severity of BE in patients [16].

In conclusion, childhood BE in is associated with increased systemic pro-inflammatory/cytotoxic T cells in the peripheral blood. While strategies that target these pro-inflammatory/cytotoxic cells might conceivably reduce future co-morbidities in these patients, further research needs to be done to more fully understand inflammation in BE.
